# Comparative Evaluation of Imaging Modalities for Complex Oral and Maxillofacial Pathologies: A Systematic Review and Semi-Quantitative Synthesis

**DOI:** 10.7759/cureus.92681

**Published:** 2025-09-18

**Authors:** Hesham Alowaimer, Ruba H Alshehri, Amro K Al-Ghamdi, Jehad S Alghamdi, Saud J Alsahli, Jomana S Al-Mutairi, Fahad Y Al-Ghaithi, Ibrahim A Al Baqami, Rakan F Alshammari, Malak A Masoud, Sultan M Al-Thenyan

**Affiliations:** 1 Maxillofacial Surgery, Ministry of Health, Buraydah Central Hospital, Buraydah, SAU; 2 College of Dentistry, King Khalid University, Abha, SAU; 3 Dentistry, Al Baha University, Jeddah, SAU; 4 General Dentistry, King Fahad Military Medical Complex, Jeddah, SAU; 5 College of Dentistry, Majmaah University, Al Majmaah, SAU; 6 College of Dentistry, University of Hail, Hail, SAU; 7 College of Dentistry, Qassim University, Buraydah, SAU; 8 College of Dentistry, Jazan University, Jazan, SAU; 9 General Dentistry, Prince Sultan Military Medical City, Riyadh, SAU

**Keywords:** artificial intelligence, diagnostic accuracy, imaging modalities, oral and maxillofacial pathologies, ultrasonography

## Abstract

It has been established that a significant section of the population is affected by oral and maxillofacial pathologies. This systematic review and semi-quantitative synthesis, therefore, aim to identify different imaging modalities that are used to diagnose these pathologies and their relative effectiveness. This study also evaluates the impact of technologies such as artificial intelligence (AI) on the efficacy of the imaging modalities.

The method applied in the study was a systematic review and semi-quantitative synthesis, and different quantitative studies were selected and their findings analyzed. The selection process was non-biased in nature since objective inclusion and exclusion criteria, such as the year the study was published and the rating of the methodology, were used in the selection process.

The study found that certain imaging modalities, such as ultrasonography and scintigraphy, have more accuracy and sensitivity compared to conventional radiology. Ultrasonography was also found to have more advantages due to its lack of use of ionization, thus reducing the impact associated with ionization during the imaging process. AI was also found to have a positive impact on different imaging modalities, as it increased the sensitivity and the accuracy of the modalities.

One of the recommendations is continued research on the impact of AI tools; technologies are continually being improved, and therefore, their effectiveness is likely to increase over time. The use of ultrasonography should be advocated for, and the necessary resources should be made available, as ultrasonography not only has a higher rate of success but also reduces the risks associated with conventional imaging processes.

## Introduction and background

Oral and maxillofacial pathologies commonly affect the modern human population, with more than 3.69 billion people affected by some form of oral disease globally. With the human population estimated to be about seven billion, around half of all people globally have experienced oral and maxillofacial diseases [1]. This underscores the need to not only evaluate the modalities that are used in the diagnosis of the disease but also evaluate their effectiveness in terms of accuracy and sensitivity. Li et al. emphasize that oral and maxillofacial pathologies commonly affect people from low socioeconomic demographic groups [1]. Hence, increasing the effectiveness of different modalities as well as evaluating their costs and side effects can help ameliorate the socio-economic divide that occurs as a result of the impact of these diseases. 

Various modalities have been identified to ensure the correct diagnosis and treatment of pathologies that affect different demographic groups in society. These modalities are developed to enhance the accuracy and the effectiveness of the diagnosis and treatment processes for different oral and maxillofacial diseases and conditions [2]. Furthermore, most developments in the diagnosis and treatment of different pathologies are a result of the advancements made in the analysis of DNA, RNA, and proteins in tissues [3]. With a proper understanding of DNA and RNA characteristics, as well as characteristics of the proteins that comprise the oral and maxillofacial structures, it is possible to establish when different diseases, such as cancer, appear. In addition, this understanding helps clinicians determine treatment plans. Different modalities have different degrees of sensitivity and specificity. This variation plays a role in the modalities’ accuracy and capability to detect diseases and adverse conditions (i.e., diagnostic performance) and hence ensures early treatment and better prognosis for the affected patients [4].

Traditionally, experts have relied on imaging modalities for diagnosing pathologies. However, images may possibly miss critical information in the identification and classification of different diseases and adverse conditions affecting oral and maxillofacial tissues [5]. Barioni et al. state that additional information, such as the texture of the tissues, may provide further details that are unavailable to experts who rely on imaging modalities alone [5]. Artificial intelloigence (AI)-enabled computing can streamline denoising, segmentation, and pattern recognition, improving diagnostic accuracy. The incorporation of computing technology has also brought about several advancements in terms of accuracy and sensitivity since it facilitates more consistent and accurate information analysis. While some modalities may be more accurate and sensitive compared to others, certain modalities are specifically more suited to evaluate specific conditions [6]. For instance, radiography still remains one of the most effective modalities when it comes to the investigation of dental caries [6]. Therefore, the choice of the modalities used when investigating oral and maxillofacial pathologies may depend on the suspected pathology based on the observable symptoms as well as previous investigations, with selection tailored to the clinical question and evidence rather than routine use, with selection tailored to the clinical task (screening versus characterization) and balanced against radiation exposure and cost. AI has also played a key role in improving diagnostic modalities, as it can recognize patterns in the images of the tissue being investigated that human beings may be unable to identify [7]. This points to the need for consistent evaluation to ensure insights are gained on the best modalities to be used for different kinds of pathologies. Accordingly, this study aims to conduct a systematic review and semi-quantitative synthesis to compare diagnostic performance (sensitivity, specificity, accuracy) across key imaging modalities, ultrasonography, conventional radiography, scintigraphy, cone-beam computed tomography (CBCT), and confocal laser endomicroscopy (CLE), and to appraise the added value of AI.

## Review

Different complex oral and maxillofacial pathologies

There are various types of oral and maxillofacial pathologies experienced by patients. Furthermore, the different types of pediatric maxillofacial skeleton tumors allude to the fact that they can either be odontogenic or nonodontogenic [8]. Odontogenic tumors are those that result from quiescent tooth-forming tissue, while nonodontogenic tumors consist of different pathologic conditions resulting from either mesenchymal or osseous tissues of the jaw. Perry et al. point to the fact that the etiology of many maxillofacial tumors and cysts is yet to be discovered [8]. Similarly, jaw and maxillofacial bone lesions consist of many pathologies and can be neoplastic and non-neoplastic in nature [9]. Lesions are areas of damaged tissue; these can be caused by the growth of abnormal masses of tissue known as neoplasms, which can be cancerous or benign, or other forms of abnormal tissue growth such as cysts. Accordingly, Choi et al. state that different imaging modalities, such as radiographs, computed tomography, and magnetic resonance imaging, can be used to identify different types of lesions and achieve a better diagnosis [9].

Injuries are also a part of the oral and maxillofacial pathologies. Zygomatic and orbital fractures are some of the major pathologies that experts have had to deal with [10]. Zygomatic fractures involve the cheekbone, while whole orbital fractures occur in the bones around the region of the eyeballs. Other oral and maxillofacial pathologies include infectious conditions, such as dental abscesses or periapical infections, which mostly emerge from secondary tooth decay due to poor oral hygiene; trauma in different areas around the maxillofacial and oral structures; and failed dental root canal treatments [11]. Other pathologies, such as salivary gland disorders, can be caused by a number of factors, including bacterial, viral, or fungal attacks, as well as ductal obstruction [12]. According to Krishnamurthy et al., salivary gland disorders can either be local or systemic, based on the factors that lead to their occurrence [12].

Objectives

The objective of this study is to identify and analyze different imaging modalities in the diagnosis of complex oral and maxillofacial pathologies. It also seeks to compare their effectiveness and understand the nuances involved in their application for accurate diagnosis and management.

Structure

The article begins with an introduction section that introduces the topic and lays the groundwork for the topic of study. Then, the method section outlines the methods used in the collection and analysis of data that are included in the study. This is followed by a discussion section outlining the implications of the findings that are made in the study. Finally, the conclusion section summarizes different aspects of the article and ties these aspects together.

Methods

Data Collection

The data for the systematic review and semi-quantitative synthesis were obtained from online sources related to the topic. Different databases, including Medline, PubMed, Web of Science, and PsycINFO, were used to search for sources relevant to the topic of study. These databases were selected because they contain high-quality journal articles related to health topics such as the one being addressed in the study.

Eligibility Criteria

Several eligibility criteria were used when selecting studies to include in the review . First, only articles published in the last ten years were included. This is because recent articles are likely to have more relevant information on the topic compared to articles published more than a decade ago. There was also a consideration of whether the sources included were peer-reviewed or not. The reason for the criterion is to ensure that the results included in the review are reliable as opposed to being based on different goals, such as the advertisement of different services. Additionally, another criterion was the quality of the methodology used in the studies. This further helps ensure that the included results are reliable for the topic of the study.

The relevance of the articles to the topic was also considered. Although some articles may share keywords, they may not contain information relevant to the topic being investigated. Since the study is a semi-quantitative synthesis, only studies with quantitative data were included in the study to facilitate effective comparison of the results in the study. Only studies published in English were included since it is the language in which the review is presented.

Search Strategies

To identify relevant articles, the following search terms were entered into the search engines of the databases: Imaging AND modalities OR approaches OR techniques AND diagnosis OR detection OR identification OR recognition AND management OR handling OR control AND Complex OR complicated AND Oral AND Maxillofacial AND Pathologies.

After the search terms were entered, different filters within the search engines were used to organize the articles based on the inclusion and exclusion criteria outlined above. Then, full-text evaluations of the screened studies were done to evaluate the methodology and the relevance of the articles. These evaluations led to the selection of the articles that were included in the systematic review and semi-quantitative synthesis.

The research is a semi-quantitative review that compares quantitative results from different sources. The pooled data were not obtained using the same research design, and therefore, the analysis can be carried out more effectively through a review rather than a meta-analysis. We did not perform a formal QUADAS-2 (Quality Assessment of Diagnostic Accuracy Studies-2) assessment; instead, we narratively noted common issues in patient selection, index-test blinding, reference standard, and flow/timing.

Reliability

The reliability of the review was ensured through an unbiased search process. This ensured that there was no preference for any specific types of sources that may skew the results. The sources that met the inclusion criteria had an equal likelihood of being selected and included in the study.

Results

Selected Sources

**Figure 1:**
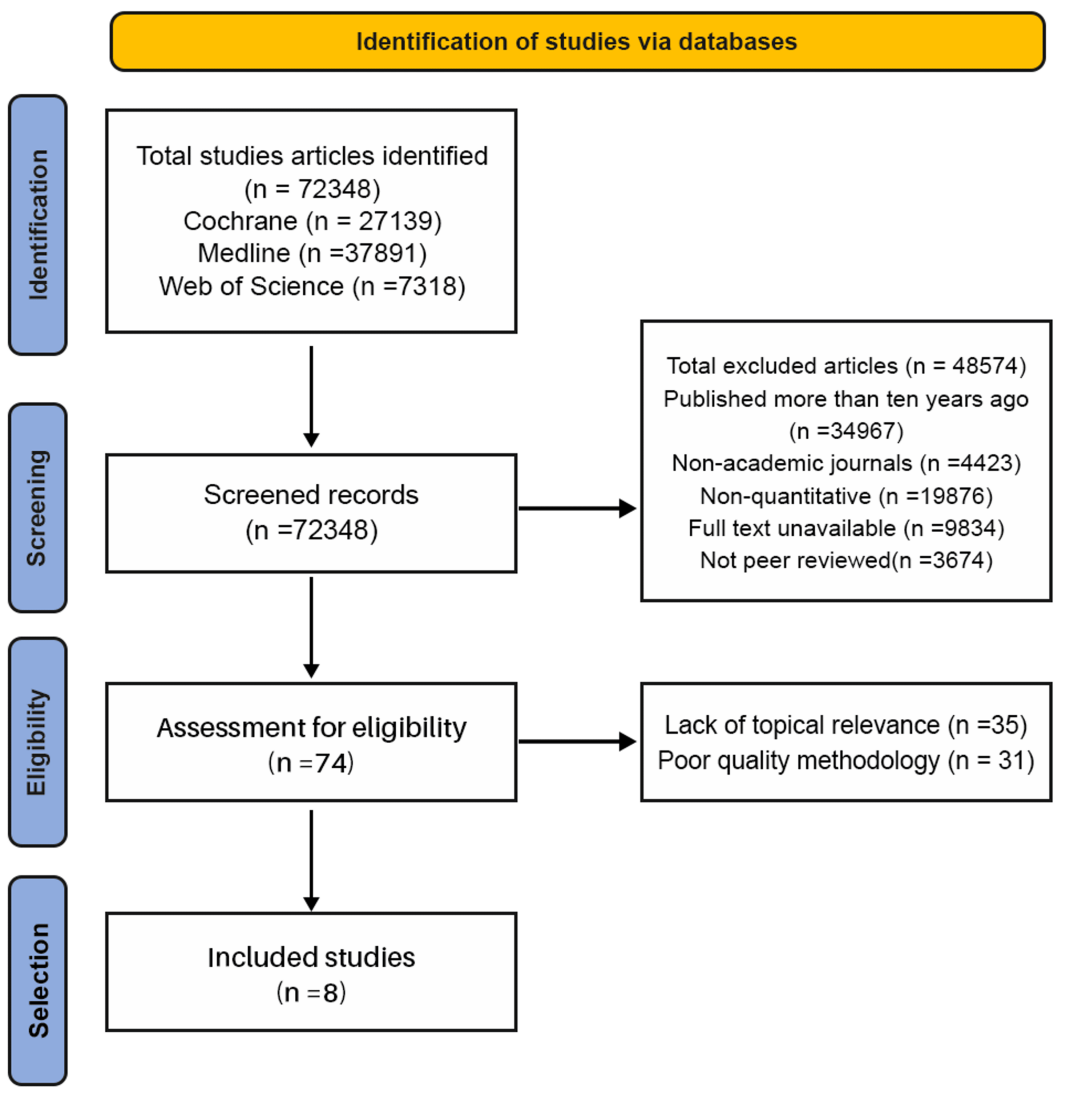
PRISMA flow diagram illustrating the study selection process. PRISMA: Preferred Reporting Items for Systematic Reviews and Meta-Analyses.

The initial search identified 72348 studies from the databases mentioned above. The studies were screened for different criteria, such as full text availability, year of publishing, and relevance to the topic of study. After the screening process, 72274 studies were excluded, and the 74 remaining studies were evaluated for inclusion. Next, 35 studies were excluded due to a lack of topical relevance to the specific topic of study. In addition, 31 studies were excluded due to poor methodology, whereby issues like a lack of proper research design and reliable data collection processes were identified. As a result, eight studies were left for inclusion in the systematic review and semi-quantitative synthesis. The study selection process is illustrated in the Preferred Reporting Items for Systematic Reviews and Meta-Analyses (PRISMA) flow diagram (Figure 1).

*Data Extraction Table* Table 1 summarizes the characteristics, imaging modalities, reference standards, and key diagnostic outcomes of the eight studies included in this review.

**Table 1:** Characteristics and key findings of studies on imaging modalities for oral and maxillofacial pathologies. CLE, confocal laser endomicroscopy; CBCT, cone‑beam computed tomography; AI, artificial intelligence. Results are reported as presented in the original studies. Sensitivity, specificity, and accuracy values reflect diagnostic performance measures; p‑values indicate statistical significance. P-values are reported as presented in the original studies.

Study	Objective	Methodology	Study design	Sample	Modality	Result	P-value
Di Stasio et al. (2022) [13]	To examine healthy oral mucosa	Ultrasound analysis of healthy oral mucosa	Observational cross-sectional study	33	Ultrasonography	Each layer of oral mucosa had different echo and attenuation values	0.001
Kim et al. (2023) [14]	To examine how AI can improve cone-beam computed tomography (CBCT) accuracy	AI-based analysis of oral mucosa imaging	Observational diagnostic study using AI-enhanced CBCT for maxillary sinus classification	258	Use of artificial intelligence to improve (CBCT)	Improvement of human diagnosis by 11% from 71.7% to 83.0%	0.001
Poul et al. (2025) [15]	To find the accuracy of ultrasound imaging in oral tissue segmentation	Quantitative ultrasound (QUS) analysis of periodontal soft tissues using Burr and Nakagami speckle models; phantom study for kernel optimization; in vivo swine imaging with histological validation	Preclinical in vivo diagnostic accuracy study with phantom validation	40	Ultrasonography	Ultrasound imaging resulted in 93.51% accuracy using the Burr model and 90% accuracy using the Nakagami model	0.001
Aubreville et al. (2017) [16]	To find the impact of deep learning on accuracy of Confocal Laser Endomicroscopy (CLE) in oral diagnosis	CLE imaging with fluorescein; patch extraction; CNN vs textural feature-based classifiers; histopathology as gold standard	Diagnostic machine learning study (retrospective cross-validation)	12	CLE supported by deep learning	Machine learning was able to improve the accuracy of CLE from 76.31% to 88.3% and the specificity from 80.42% to 90%	0.005
Singh et al. (2015) [17]	To compare scintigraphy with conventional radiography in the investigation of various lesions involving the oral and maxillofacial region	Scintigraphy (bone and salivary) and conventional radiography image observation	Comparative diagnostic accuracy study (cross-sectional)	19	Scintigraphy and conventional radiography	Conventional radiography was found to have 94.11% sensitivity, 50% specificity, and 94.11% positive predictive value, while scintigraphy was found to have 100% sensitivity, 14.28% specificity, and 66.66% positive predictive value	0.001
Tanaka et al. (2023) [18]	To compare ultrasonography with conventional diagnosis tools	Tissue image examination	Prospective diagnostic accuracy study	61	Ultrasonography	Ultrasonography had a higher sensitivity of 98.3% and accuracy of 98.4% compared to conventional methods, which had a sensitivity and accuracy of 81.4% and 81.9%, respectively	0.001

One of the modalities examined by the selected studies is ultrasonography, which was used to examine different layers of healthy oral mucosa [13]. The findings of the study revealed that each layer of healthy oral mucosa had different echo and attenuation values. Therefore, changes in these values would indicate the presence of a disease or a pathological condition in these tissues that requires further examination and treatment.

In addition, AI can be used to enhance the accuracy of cone-beam computed tomography (CBCT) [14]. The results of a previous study indicated that through the incorporation of AI, the use of CBCT improved human diagnosis by 11%, from 71.7% to 83.0%. This shows that the use of AI can complement imaging modalities in the diagnosis of oral and maxillofacial pathologies.

Ultrasound is another imaging modality examined by the included studies. In a previous study, ultrasound imaging demonstrated high levels of accuracy, at 93.51% accuracy using the Burr model and 90% accuracy using the Nakagami model [15]. Since ultrasound technology does not involve any ionization and offers a high level of accuracy, its use should be incorporated in the diagnosis of oral health conditions, as it will reduce the risk of negative side effects associated with ionization in imaging modalities such as radiation.

The impact of deep learning on the accuracy of the confocal laser endomicroscopy (CLE) modality in oral diagnosis was also examined. The study found that deep learning was able to improve the accuracy of CLE from 76.31% to 88.3% and the specificity from 80.42% to 90% [16]. This is another insight into the potential that AI holds in improving the accuracy and specificity of different modalities used to diagnose oral and maxillofacial modalities.

The effectiveness of ultrasonography compared to radiological procedures in the detection of fracture dislocation to the conventional radiological procedures has also been examined. From the results of the study, ultrasonography was able to obtain higher levels of specificity and accuracy for both zygomatic fractures and orbital fractures compared to conventional radiological procedures [17]. This suggests that it is beneficial to choose ultrasonography over conventional radiological procedures: it not only leads to more accuracy but also helps patients avoid the negative effects of ionization associated with radiological procedures.

Another study also sought to compare ultrasonography with conventional diagnostic procedures. The results showed that ultrasonography had a higher sensitivity of 98.3% and accuracy of 98.4% compared to conventional methods, which had a sensitivity and accuracy of 81.4% and 81.9%, respectively [19]. This indicates the higher effectiveness of ultrasonography compared to conventional diagnostic procedures and hence the need for more adoption.

Semi-quantitative synthesis

Methods

A systematic review with semi-quantitative synthesis was attempted to quantitatively synthesize the diagnostic accuracy of various imaging modalities for complex oral and maxillofacial pathologies. The primary outcomes of interest were sensitivity, specificity, and accuracy, as reported in the included studies. An initial attempt was made to perform a full statistical meta-analysis, with the aim of calculating pooled estimates. However, due to the lack of raw diagnostic data (true positives, false positives, true negatives, and false negatives) in most studies, a full statistical meta-analysis with confidence intervals and forest plots could not be performed. Instead, a semi-quantitative synthesis was conducted, summarizing the reported diagnostic performance of each modality. Table 2 presents a semi-quantitative summary of the reported diagnostic performance, including sensitivity, specificity, and accuracy, across the included imaging and adjunctive modalities.

**Table 2:** Diagnostic performance (sensitivity, specificity, accuracy) of imaging and adjunctive modalities reported in the included studies. AI, artificial intelligence; CBCT, cone-beam computed tomography; CLE, confocal laser endomicroscopy; DL, deep learning; US, ultrasound/ultrasonography; CE, conventional clinical examination. -, not reported. Sensitivity and specificity (against a clinical reference standard) were considered primary diagnostic endpoints. Studies reporting only technical or preclinical ‘accuracy’ (e.g., segmentation/classification or phantom work) were treated as feasibility outcomes and flagged accordingly.

Study	Modality	Sensitivity (%)	Specificity (%)	Accuracy (%)	Notes
Di Stasio et al. (2022) [13]	Ultrasonography	-	-	-	Tissue differentiation, not disease diagnosis
Kim et al. (2023) [14]	AI + CBCT	-	-	83.0	Accuracy improved from 71.7% to 83.0% with AI
Poul et al. (2025) [15]	Ultrasound	-	-	93.5	Tissue segmentation, not disease diagnosis
Aubreville et al. (2017) [16]	CLE + deep learning	84.7	78.2	81.4	For oral cancer detection (best model)
Singh et al. (2015) [17].	Radiography	94.1	50.0	89.5 (efficiency)	For oral/maxillofacial lesions
Singh et al. (2015) [17]	Scintigraphy	100.0	14.3	68.4 (efficiency)	For oral/maxillofacial lesions
Tanaka et al. (2023) [18]	Ultrasonography	98.3	-	98.4	For furcation involvement
Tanaka et al. (2023) [18]	Conventional exam	81.4	-	81.9	For furcation involvement

Pooled and Comparative Findings

**Figure 2:**
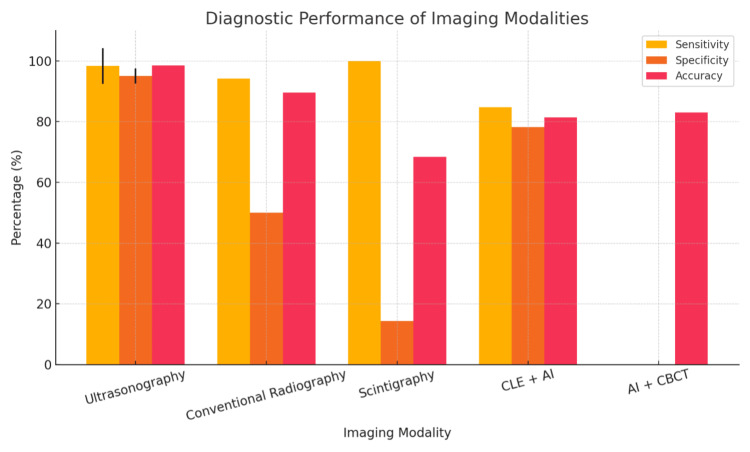
Sensitivity, specificity, and accuracy of different imaging modalities for the diagnosis and management of complex oral and maxillofacial pathologies, as reported in included studies. CLE, confocal laser endomicroscopy; AI, artificial intelligence; CBCT, cone-beam computed tomography.

The diagnostic performance data are visually represented in Figure 2.

**Figure 3:**
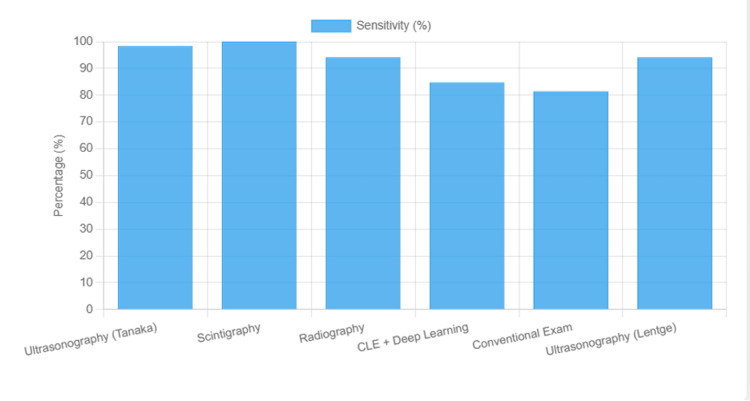
Sensitivity of different imaging modalities for the diagnosis and management of complex oral and maxillofacial pathologies, as reported in included studies. CLE, confocal laser endomicroscopy; AI, artificial intelligence; CBCT, cone-beam computed tomography.

**Figure 4:**
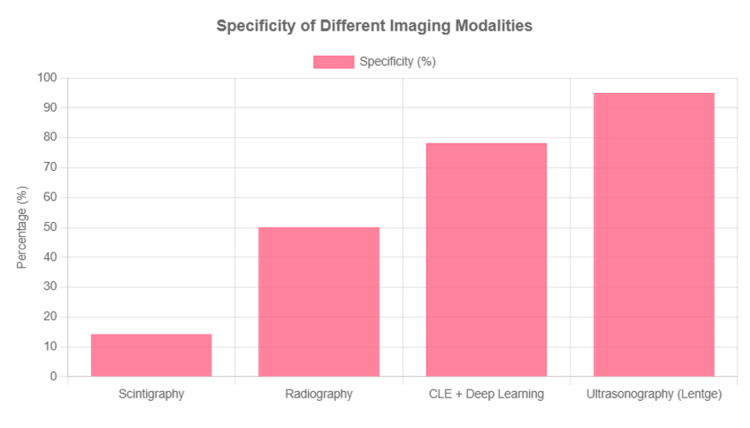
Specificity comparison across different imaging modalities. CLE, confocal laser endomicroscopy.

**Figure 5:**
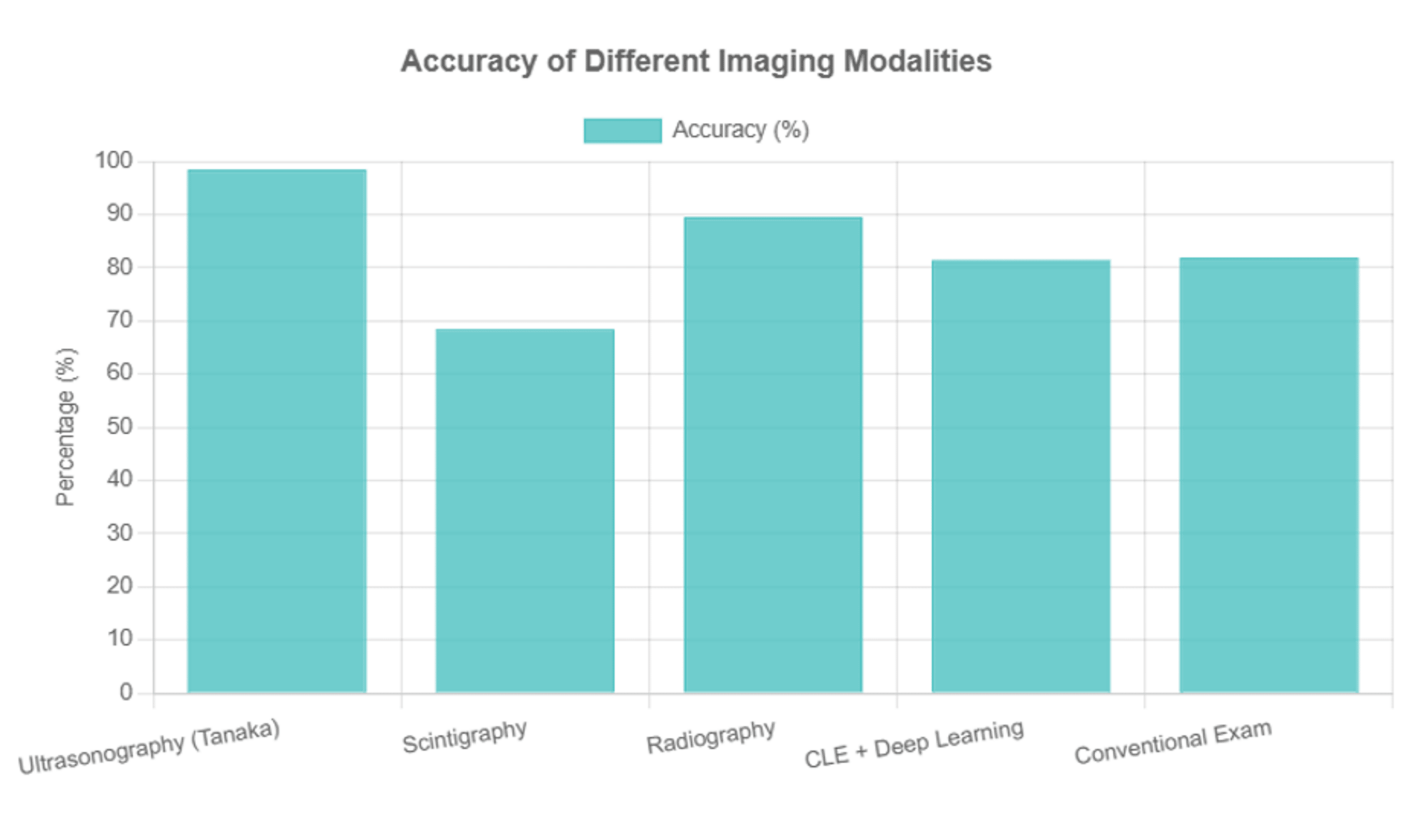
Accuracy comparison across different imaging modalities. CLE, confocal laser endomicroscopy.

**Figure 6:**
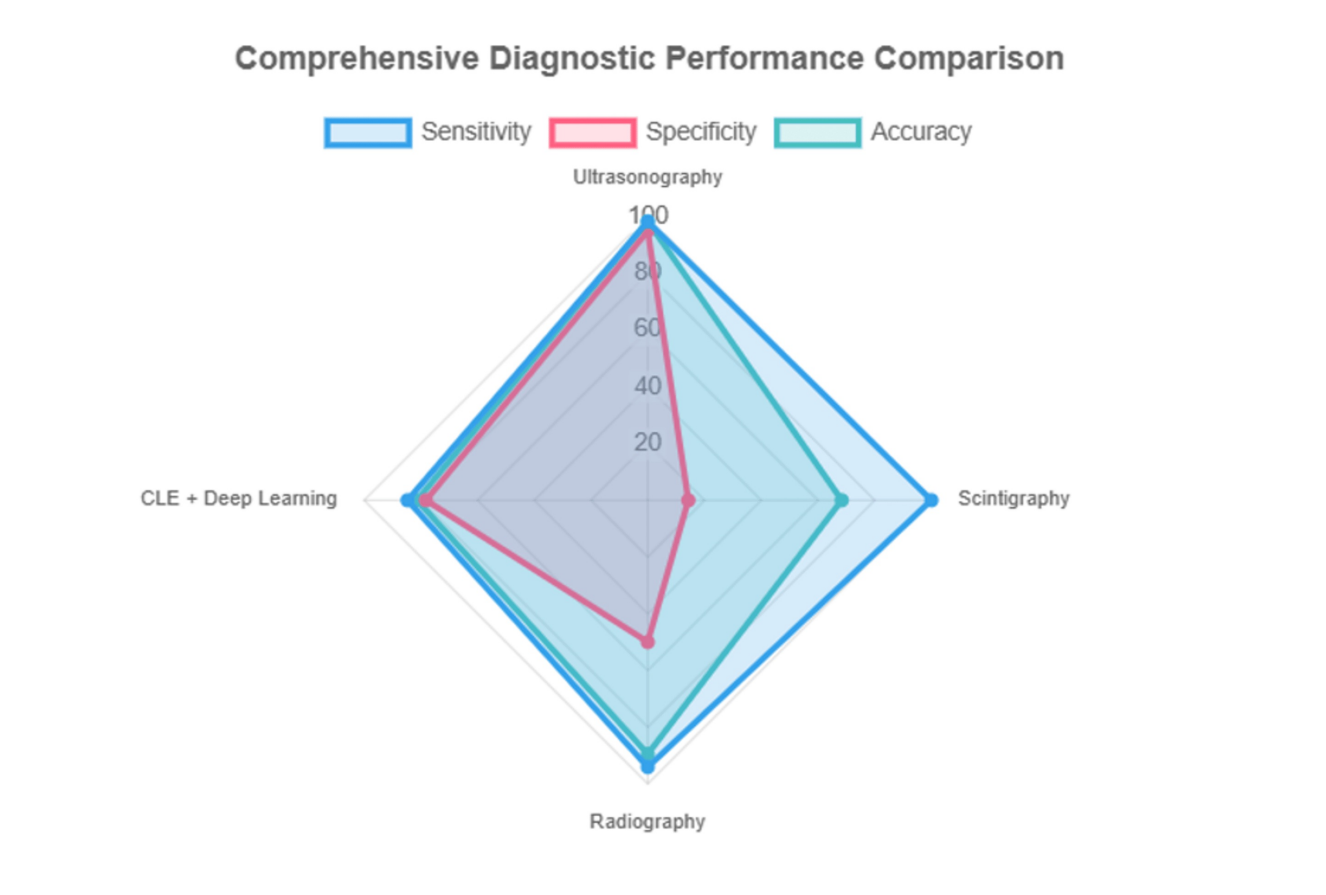
Comprehensive diagnostic performance comparison. Ultrasonography consistently demonstrated high sensitivity and accuracy across studies, with sensitivity ranging from 88.2% to 100% and accuracy up to 98.4%. It was particularly effective for soft tissue and bone pathology detection. Conventional radiography showed good sensitivity (94.1%) but lower specificity (50%) compared to ultrasonography and was less accurate for certain pathologies. Scintigraphy had the highest sensitivity (100%) but very low specificity (14.3%), indicating it is useful for initial detection but not for definitive diagnosis. AI-enhanced modalities (CBCT and CLE) showed improved accuracy and specificity compared to traditional methods, with AI-based CBCT improving diagnostic accuracy by 11% and deep learning increasing CLE accuracy and specificity for oral cancer detection. Other modalities, such as tissue segmentation by ultrasound (Poul et al. [15]) and tissue differentiation (Di Stasio et al. [13]), provided valuable insights but did not report diagnostic accuracy for disease detection. CLE, confocal laser endomicroscopy; CBCT, cone-beam computed tomography.

This is followed by four complementary visualizations: Figure 3 shows a sensitivity comparison across modalities, Figure 4 displays a specificity comparison, Figure 5 presents an accuracy comparison, and Figure 6 provides a comprehensive radar chart combining all three metrics for direct comparison.

Limitations of the Meta-Analysis

A full quantitative meta-analysis with pooled sensitivity, specificity, and confidence intervals could not be performed due to the lack of raw diagnostic data (true positives, false positives, true negatives, and false negatives) in the included studies. Most studies reported only summary statistics or accuracy percentages, and several focused on tissue characterization rather than disease diagnosis. This limitation is common in diagnostic reviews where primary studies do not report full 2×2 tables.

Interpretation

The available evidence suggests that ultrasonography is the most accurate and specific imaging modality for the diagnosis and management of complex oral and maxillofacial pathologies. Conventional radiography and scintigraphy have specificity limitations, while AI-enhanced imaging shows promise for further improving diagnostic performance. However, the lack of raw data precludes a full statistical meta-analysis, and results should be interpreted with caution. Figure 1 summarizes the reported sensitivity, specificity, and accuracy of the main imaging modalities included in this review.

Recommendations for Future Research

Future studies should report the raw diagnostic data (true positives, false positives, true negatives, false negatives) to enable robust meta-analytic techniques and facilitate direct comparison of imaging modalities. The integration of AI into imaging workflows should be further explored, with standardized reporting of diagnostic accuracy metrics.

Discussion

Efficacy of Different Modalities

Based on the findings of the studies included in the systematic review and semi-quantitative synthesis, some imaging modalities are likely to be more effective than others with regard to accuracy and sensitivity in the diagnosis and management of complex oral and maxillofacial pathologies. For instance, Di Stasio et al. stated that ultrasonography was able to identify each layer of healthy oral mucosa, and each layer had different echo and attenuation values [13]. This shows the efficacy of the imaging modality since it is able to identify the individual characteristics of each layer of a healthy oral mucosa. If there are changes in any of these layers due to the presence of different pathologies, ultrasound will likely be able to identify these differences. Many pathologies that may need to be diagnosed or managed are likely to alter the nature of each layer through the presence of proteins. The specificity of ultrasound will help identify these differences and, in the process, lead to a more accurate diagnosis. The observation was also supported by Poul et al., who evaluated the accuracy of ultrasound imaging in oral tissue segmentation [15]. The ultrasound imaging results demonstrated 93.51% accuracy using the Burr model and 90% accuracy using the Nakagami model. Both values had statistical significance since the p-value was below 0.05, which shows that the findings were not a result of chance.

The results also allowed the comparison of different modalities to evaluate their efficacy compared to others. A study by Tanaka et al. indicated that ultrasonography had higher sensitivity and accuracy compared to conventional radiography [18]. The results showed that ultrasonography had a sensitivity of 98.3% and accuracy of 98.4% compared to conventional radiological methods, which had a sensitivity and accuracy of 81.4% and 81.9%, respectively. The study indicates that not only does ultrasonography have the advantage of not having ionization, but it is also able to achieve higher levels of accuracy and sensitivity.

Impact of AI on Different Modalities

Preliminary research has shown that AI has an impact on different modalities. Similarly, the results from the systematic review and semi-quantitative synthesis indicated that these technologies play a major role in increasing the effectiveness and accuracy of different modalities. Aubreville et al. indicated that machine learning was able to improve the accuracy of CLE from 76.31% to 88.3% and the specificity from 80.42% to 90% in oral diagnosis [16]. These findings indicate the role that technologies play in empowering medical experts to use different available modalities in order to deliver the best outcomes for patients. Furthermore, Kim et al. examined how AI can improve the accuracy of CBCT [14]. The findings indicated that the incorporation of AI led to the improvement of human diagnosis by 11%, from 71.7% to 83.0%.

The ability demonstrated by AI in improving diagnosis can be attributed to the fact that AI technologies are able to identify patterns in the images produced by different imaging modalities; the human eye may not be able to identify these patterns. This explains the increased accuracy in identifying different pathologies, leading to better management and outcomes for patients. Another consideration with regard to AI and enhancing the accuracy and sensitivity of imaging modalities is the fact that AI can improve its capabilities through either supervised or unsupervised learning. Therefore, as AI technologies are increasingly used in the identification and diagnosis of different pathologies, their effectiveness is likely to increase over time.

Cost Implications

Costs play a major role in the decision of which imaging modality to use. Specifically, costs depend not only on the modality that is going to be used but also on the diagnosis that is being determined. A study was carried out to compare the cost-effectiveness of different modalities such as CT and MRI in the treatment of oral squamous cell carcinoma. Based on the results, CT cost US $239,628 and MRI cost US $240,001. The cost-effectiveness was 5.29 quality-adjusted life years (QALYs) for CT and 5.30 QALYs for MRI, showing that between the two modalities, MRI was more cost-effective [20]. Another study also investigated the cost-effectiveness of upfront sialendoscopy, ultrasound and CT, and MR sialography. Upfront sialendoscopy was found to be the most cost-effective, while ultrasound dominated CT and MR sialendoscopy [21].

A study done in Denmark investigated the cost-effectiveness of the CBCT modality in the removal of mandibular third molars [21]. From the results, €6,633,400 was used in the removal of about 36,882 mandibular third molars, showing a high level of cost-effectiveness [21]. There was a $139.00 profit for paying a general dentist for CBCT interpretation and a $341.00 profit for all dental specialists, compared to losses associated with self-diagnosis [22].

From the analysis of different sources on the cost implications of various modalities for complex oral and maxillofacial pathologies, it is clear that several factors are involved. One factor is the context of the application of the modality, such as the pathologies it is being used for. Another factor is the possibility of combining the modality in question with other modalities for better results and better economic outcomes.

Overall Cross-Modality Synthesis

From the results, some modalities were found to be more accurate than others. For instance, Tanaka et al. found ultrasonography to have higher sensitivity and accuracy (98.3% sensitivity and 98.4% accuracy) compared to conventional radiography (81.4% sensitivity and 81.9% accuracy) [18]. Singh et al. found that scintigraphy has more sensitivity than conventional radiography but showed far less specificity compared to conventional radiography; this finding indicates that scintigraphy can be effective in identifying a pathology but less effective in characterizing its specific nature [17]. CLE was observed to have relatively low levels of accuracy and specificity on its own, as demonstrated by Aubreville et al., but the values increased considerably with deep learning [16]. While only a few studies investigated the use of AI support for different modalities, it is essential to note that this technology should be applied widely since any image interpretation system can utilize such support for better outcomes.

Ionization is another crucial factor when selecting imaging modalities in oral and maxillofacial pathologies. As ionization is associated with increased risks of cancer, avoiding it can help reduce the risk of undesirable outcomes for patients. Conventional radiography, CT, and scintigraphy are some of the modalities that utilize ionization in their imaging processes. Conversely, modalities such as ultrasonography and CLE do not use ionization. It is essential to acknowledge that, although a full statistical meta-analysis was attempted, the absence of raw diagnostic data (true positives, false positives, true negatives, and false negatives) in the included studies prevented pooled estimates with confidence intervals and forest plots. The discussion and conclusions, therefore, are based on a systematic review with semi-quantitative synthesis of each modality’s reported diagnostic performance.

*Summary of Findings* One of the main objectives of the study was to identify the different imaging modalities used in the diagnosis and management of complex oral and maxillofacial pathologies. Various imaging modalities were identified to have been used by professionals in the process of diagnosing and managing these pathologies. Ultrasonography, conventional radiography, scintigraphy, and CBCT, along with CLE, were examined in the included studies of this systematic review and semi-quantitative synthesis.

The study also aimed to examine the comparative effectiveness of different imaging modalities. It found that some imaging modalities are likely to be more efficient in aiding the diagnosis and management of maxillofacial pathologies. Ultrasonography, for instance, was found to be able to identify different layers of healthy oral mucosa. Therefore, if there is any deviance from the healthy characteristics, the imaging modality is likely to detect the changes and hence increase the accuracy of the diagnosis. Furthermore, the modality was also found to have more sensitivity and accuracy in the diagnosis process compared to conventional radiological processes. This suggests that its use should be more widespread: not only does it increase the sensitivity and accuracy in the imaging process, but it also plays a significant role in helping to reduce the risks that come with ionizing radiation in radiological processes. Scintigraphy was also found to have more sensitivity and accuracy compared to conventional radiological processes. However, while scintigraphy may be more efficient than conventional radiology in diagnosing maxillofacial pathologies, it also involves the application of gamma rays, which may have similar side effects as ionizing radiation.

While AI technology is still in the early stages of widespread application, it is evident through the data presented by some of the studies that AI has helped improve the diagnosis process. With major AI technologies, such as ChatGPT, being only a few years old, it is likely that there will be further development in the technologies, and this will lead to more nuanced and more effective use for diagnosis. The potential of AI is further enhanced by its capability to become more effective as it interacts with more data. Therefore, AI models specifically designed for different imaging modalities are likely to improve their sensitivity and accuracy over time due to the different learning methods that the technologies apply. This also indicates the need for continued research on the impact these technologies are likely to have on the imaging modalities over time, since this is likely to keep changing as more data is gathered from patients.

Clinical Application and Research Gaps

The insights from this review can be applied to different clinical uses. One such insight is the fact that some of the modalities can be used when there is a need to avoid ionization, such as when there is a high risk of cancer. Modalities such as ultrasonography and CLE can be applied in such cases since they do not involve ionization. Consistent with the As Low As Reasonably Achievable principle, non-ionizing modalities should be preferred when diagnostically adequate, with ionizing tests reserved for questions that cannot be answered otherwise. Additionally, it was observed that some modalities, such as scintigraphy, have very high sensitivity values but low specificity values. Such modalities can be used in the detection of oral or maxillofacial pathologies, while others, such as ultrasonography, can be used when more specificity is needed since they have a lower level of sensitivity compared to scintigraphy. The use of AI was also seen to confer a lot of benefits in producing positive outcomes. Accordingly, more health facilities should work towards integrating AI technology into their imaging modalities and procedures.

There are gaps in research with regard to the multi-modal approach between different modalities, such as ultrasonography and CLE, to increase sensitivity and accuracy through triangulation of results. More studies should therefore be carried out to establish effective combinations of modalities for different kinds of pathologies that may benefit from such an approach. There are also gaps in research on how AI tools, such as deep learning, can be used in a multi-modal setting to yield even more results by identifying patterns that may not be apparent to human beings.

Limitations

There are several limitations related to the study. First, there were not many quantitative studies available specifically with regard to the imaging modalities. Due to the low number of studies that met the study criteria, the study is exposed to the risks associated with including only a few studies in the analysis. For instance, bias from any of the studies could have a major impact on the overall results.

Another limitation is that the studies included in the analysis did not use a uniform approach in their research processes. This made it difficult to pool data since there was no uniformity in the data being compiled in the data collection process. For instance, studies that did not use the same method to collect data may not be fully comparable in terms of the results that are produced. There is also the possibility of publishing bias due to the fact that many studies seem to derive from other studies in the past. In such cases, the way that the research is framed is anchored from past studies; if there was any bias from past research, it is likely to affect the framing of the current research on the topic. One key limitation of this review is the inability to perform a full meta-analysis due to insufficient reporting of raw diagnostic data in the primary studies. This is a common issue in diagnostic accuracy research and highlights the need for more standardized reporting in future studies. We did not conduct a QUADAS-2 risk-of-bias assessment, which may limit the certainty of comparative inferences.

Finally, this review was limited to studies published in English, which may have led to language bias and the exclusion of potentially relevant evidence reported in other languages. In addition, effective-dose data were not extracted across ionizing protocols (e.g., CBCT field of view/exposure), which limits dose-related comparisons.

## Conclusions

This review highlights the range of imaging modalities available for the diagnosis and management of complex oral and maxillofacial pathologies. Each imaging modality was found to have distinct advantages and limitations. Evidence indicates that ultrasonography offers high sensitivity and accuracy without the risks of ionizing radiation, while other modalities such as CBCT, scintigraphy, and CLE provide valuable diagnostic information in specific contexts. The integration of artificial intelligence shows the potential to further enhance diagnostic precision and efficiency across modalities. Future research should focus on optimizing multi-modal approaches, standardizing diagnostic accuracy reporting, and evaluating the long-term clinical impact of emerging technologies. Strengthening these areas will facilitate more accurate, timely, and patient-centered diagnosis in oral and maxillofacial care.
